# Association of two geriatric treatment systems on care home admission and mortality in patients with hip fracture

**DOI:** 10.1186/s12877-022-03037-z

**Published:** 2022-05-27

**Authors:** Kilian Rapp, Clemens Becker, Chris Todd, Martin Rehm, Dietrich Rothenbacher, Claudia Konnopka, Hans-Helmut König, Thomas Friess, Gisela Büchele

**Affiliations:** 1 Department of Clinical Gerontology, Robert-Bosch-Hospital, Auerbachstr.110, 70376 Stuttgart, Germany; 2 School of Health Sciences, Faculty of Biology, Medicine and Health, The University of Manchester, Manchester, M13 9PL UK; 3 Manchester University NHS Foundation Trust, Manchester, M13 9WL UK; 4 Institute of Epidemiology and Medical Biometry, Ulm University, Ulm, Germany; 5 Center for Trauma Research, Ulm University, Ulm, Germany; 6 Department of Health Economics and Health Services Research, University Medical Center Hamburg-Eppendorf, Hamburg, Germany; 7 AUC - Akademie der Unfallchirurgie GmbH, Wilhelm-Hale-Straße 46b, 80639 München, Germany

**Keywords:** Hip fracture, Geriatric, Rehabilitation, Care home admission, Mortality

## Abstract

**Background:**

In Germany, geriatricians deliver acute geriatric care during an acute hospital stay and subacute rehabilitation after transfer to a rehabilitation clinic. However, the proportion of patients who receive acute geriatric care (AGC) or are transferred to subacute rehabilitation (TSR) differs considerably between hospitals. The aim of this study was to analyse the association between the two geriatric treatment systems and care home admission or mortality in patients following hip fracture.

**Methods:**

Health insurance claims data were used to identify the rate of AGC per hospital and the rate of TSR per hospital following hip fracture surgery in patients aged ≥ 80 years. Outcomes were cumulative admission to a care home and cumulative mortality within 6 months after hospital admission.

**Results:**

Data from 23,046 hip fracture patients from 561 hospitals were analysed. The rate of AGC was not associated with care home admission. However, compared to high rates of AGC medium rates or no AGC were associated with increased death rates by 12% or 20%, respectively. Treatment in hospitals with low rates of TSR was associated with a 8% higher risk of care home admission and a 10% increased risk of death compared to treatment in hospitals with high rates of TSR.

**Conclusions:**

Our study suggests potential effects of geriatric treatment: reduction of mortality in hospitals with high rates of AGC or reduction of care home admission and mortality in hospitals with high rates of TSR.

## Background

Hip fracture is associated with increased mortality, loss of independence and care home admission [1, 2]. Most hip fractures occur in old and frail people [3, 4]. Therefore, geriatric teams are increasingly involved in the treatment of patients with hip fractures [5].

In Germany, geriatricians deliver both ‘acute geriatric care’ (AGC) during the acute hospital stay and subacute geriatric rehabilitation after ‘transfer to a subacute rehabilitation’ clinic (TSR). AGC in patients with hip fracture is delivered in cooperation with orthopedic surgeons as orthogeriatric comanagement. It begins soon after surgery and lasts at least 14 days due to the current DRG definition. An accelerated discharge would lead to a drastic loss in reimbursement. This is acknowledged as an artefact but a widely accepted practice. The geriatric treatment is provided by a multidisciplinary geriatric team led by a geriatrician and made up of physiotherapists, occupational therapists, specifically trained nurses, social workers, and additional disciplines if needed. It includes a standardized comprehensive geriatric assessment, regular interdisciplinary team meetings, development of a rehabilitation plan with setting of functional goals and a medical focus on geriatric syndromes. Early mobilisation is a key component of the treatment procedure. In patients with hip fracture, AGC can be delivered on an orthopedic or a geriatric unit. In Germany, the two most common models are either shared responsibility on an orthogeriatric unit (integrated model) or a geriatric liaison service on the orthopedic unit with a rapid transfer to an acute geriatric unit (sequential model).

An alternative model or sometimes additional treatment is delivered in subacute geriatric rehabilitation units. Hip fracture patients are usually transferred to a subacute geriatric rehabilitation clinic after an acute hospital stay of 7 to 14 days. These patients require a pre-defined functional status, which excludes patients with a very low functional status, e.g. a Barthel Index < 40. The subacute rehabilitation lasts usually 3 weeks. Sometimes it can be extended by 1 or 2 weeks. The rehabilitation is based on a comprehensive geriatric assessment and is also provided by a multidisciplinary geriatric team. Regular interdisciplinary team meetings, development of a rehabilitation plan with setting of functional goals and a medical focus on geriatric syndromes are basic parts of the rehabilitation process.

In the past, most hospitals offered either one or the other type of geriatric treatment according to the federal state in which they were located. Since 2012 this historical situation has changed considerably. Many hospitals from states offering ‘subacute geriatric rehabilitation’ established AGC whilst the states offering AGC were required by law to develop subacute geriatric rehabilitation services. During this transition period hip fracture patients were treated at their acute stay in hospitals which delivered a) exclusively AGC or b) exclusively TSR or c) both AGC *and* TSR. But if a hospital offered AGC or TSR did not naturally mean that all hip fracture patients actually received one or even both types of geriatric treatment. Potential reasons were, that the patient’s functional status may not have qualified for the specific geriatric treatment, the (personal) infrastructure of the hospital may not have been sufficient to offer AGC to all patients, subacute geriatric rehabilitation clinics may not have been available in the nearer region and transferring hip fracture patients to subacute geriatric rehabilitation clinics may not have been a tradition in some hospitals so far. Therefore, the rates of AGC or of TSR per hospital were clearly below 100% and differed from hospital to hospital considerably.

A comparison of the different geriatric treatment systems on health-related outcomes like care home admission or mortality has rarely been undertaken. In patients with hip fracture, rates of care home admission or mortality may reflect (achieved) functional status or hospital’s quality of care and are regarded as outcomes of high relevance.

The heterogeneous situation of geriatric treatment in Germany can now be regarded as a ‘natural experiment’. We used this ‘natural experiment’ and performed a study based on health claims data to analyse the relationship between the two geriatric treatment systems on care home admission and mortality in patients aged ≥ 80 years with hip fracture.

## Methods

### Data source and study population

The basic dataset consisted of 87,022 patients aged ≥ 65 years admitted to hospitals with a femoral fracture between 01.01.2015 and 30.06.2016 in Germany and insured by the “Allgemeine Ortskrankenkasse” (AOK) insurance company. AOK is Germany’s largest health insurance company and covers nearly one-third of Germany’s 82.5 million population. Patient-related health insurance claims data were provided by the scientific institute of the AOK (“Wissenschaftliches Institut der AOK”, WIdO) in de facto anonymized manner.

**Fig. 1:**
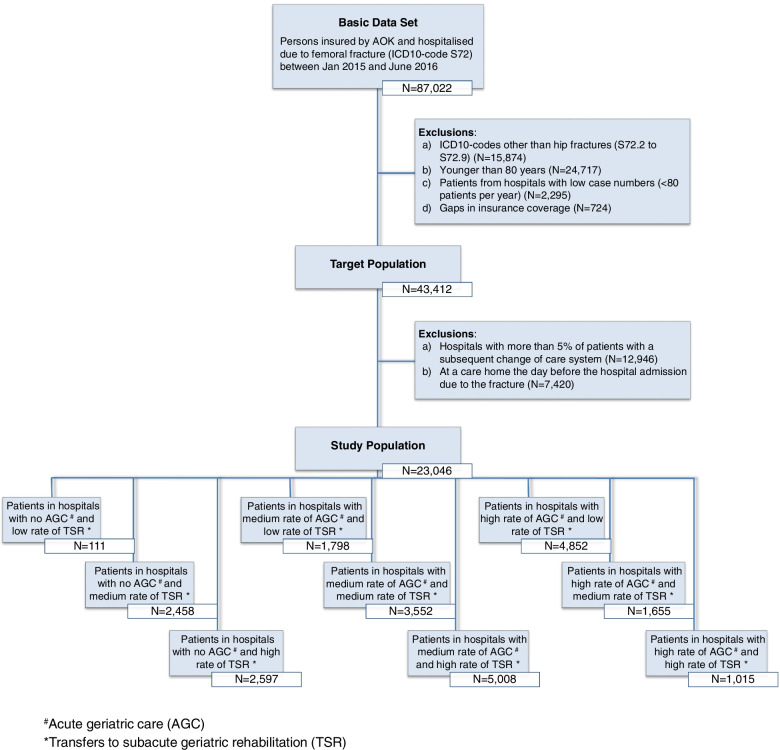
Study population and analysed groups of combinations of rates per hospital of ‘acute geriatric care’ (AGC) and ‘transfers to subacute geriatric rehabilitation’ (TSR). # Rate of acute geriatric care (OPS 8–550): None: 0%; Medium: > 0 to ≤ 48.8%; High: > 48.8%. * Rate of transfers to subacute rehabilitation: Low: < 18.6%; Medium: 18.6 to 43.6%; High: > 43.6%

Hip fractures were identified using hospital admission diagnostic codes S72.0 and S72.1 (ICD-10) (Fig. 1). Analyses were restricted to patients aged ≥ 80 years since they are by definition regarded as geriatric patients due to their age-specific vulnerability, which means increased risks of complications, chronic courses and loss of autonomy [6]. To exclude a quality-related source of heterogeneity, hospitals which treated small numbers of hip fracture patients (*n* < 80 per year) were excluded. Furthermore, patients who were residents of a care home before hospital admission were excluded.

Acute geriatric care (AGC) can be identified in insurance health claims data by the German procedure classification code ‘OPS8-550’. It represents a complex treatment of early rehabilitation lasting at least 14 days (see Table 1 for details). In patients with hip fracture, AGC begins soon after surgery and can be delivered as orthogeriatric co-management on an orthopedic or a geriatric unit.

Some orthopedic units which do not offer AGC transfer hip fracture patients early after surgery to other hospitals which provide AGC. Hospitals with such transfer exceeding 5% of their hip fracture patients were excluded from the analyses to avoid a) misclassification of exposure and b) selection bias of transferred (and not transferred) patients (Fig. 1).

After the acute hospital stay patients can be transferred to a subacute rehabilitation clinic (TSR). This information is recorded in routine hospital datasets permitting calculation of the rate of TSR per hospital. Subacute geriatric rehabilitation needs an upfront approval by the health insurance, is delivered over a time period of 3 weeks and is sometimes extended by 1 or 2 weeks (see Table 1 for details).

Independent variables were 1.) the rate of AGC (OPS 8–550) per hospital and 2.) the rate of TSR per hospital; calculated as the number of hip fracture patients with an AGC (or a TSR) in each hospital divided by the total number of hip fracture patients in the same hospital. Each variable was categorized in three groups: none (0%), medium (> 0 to ≤ 48.8%) and high (> 48.8%) rates of AGC, and low (< 18.6%), medium (18.6 to 43.6%) and high (> 43.6%) rates of TSR. For AGC the chosen categories followed a dichotomization (medium and high), for TSR a tercile split.

The OPS8-550 procedure code and information about transfers to a subacute rehabilitation clinic were used to characterize the degree to which hospitals provided AGC or TSR for their patients. Whilst, at first sight, it might seem straightforward to base analysis on individual-level data based directly on the presence or absence of OPS8-550 codes or the information about a transfer to a subacute rehabilitation clinic in individuals’ claims records, this approach is misleading and therefore not appropriate. Reasons are that a) individual allocation on the basis of OPS8-550 coding would introduce immortal time bias (survivorship bias) [7] since to be coded OPS8-550 the patient must receive a minimum of 14 days AGC, and b) that individual allocation on the basis of transfers to a subacute rehabilitation clinic would introduce a strong selection bias since subacute rehabilitation is based on functional condition. Identifying the hospitals’ rate of AGC and TSR overcomes this problem. Thus, hospitals’ geriatric health service processes in general were used to analyse the influence of different types of geriatric treatment on outcomes on a systemic level.

### Dependent variables

The outcome was cumulative admission to a care home within 6 months following hospital admission due to a hip fracture. Since AGC (qua surrogate of orthogeriatric co-management) is associated with reduced mortality [8, 9] different survival rates may influence the rate of care home admission and can therefore influence the risk for care home admission. Thus, cumulative mortality within 6 months after hospital admission was also calculated with censoring in case of a care home admission and reported as an additional outcome.

### Covariates

Age in years at the index fracture and sex were documented in the claims database. An assessment of the degree of care need is a requirement for those identified as “frail care recipients “ under German Social Security Code XI (‘*Sozialgesetzbuch’*). In order to claim for long-term care benefit, people must need daily a minimum of 90 min of assistance with basic activities of daily living such as washing, eating, or dressing and of instrumental activities of daily living such as cleaning or shopping. Verification and assessment of care need is performed by the medical service of the health insurance funds. Depending on the extent of care required recipients are categorised into 3 degrees of care need. This classification of care need can be used as a surrogate marker of disability [10]. The number of hip fracture patients per hospital per year in our dataset was used as a surrogate for the size and/or the expertise of the trauma surgery unit. Time to surgery has long been known to be associated with mortality [11, 12]. Therefore, time from hospital admission to surgery was determined and used as a covariate. Because of the link to reimbursement, the coding of comorbidities seemed to be particularly comprehensive in patients with an OPS8-550 procedure. Therefore, common comorbidity scores based on diagnoses might bias the results. Instead, a medication-based co-morbidity score was applied [13]. Prescriptions of medications were coded for the quarter before fracture and if at least one of the prescribed medications belonged to one of the 22 pre-defined disease groups, the co-morbidity counter was increased by one score-point.

### Statistical analysis

Baseline characteristics were described by means and standard deviations or absolute numbers and percentages, as appropriate. Cumulative incidences were calculated for study outcomes occurring within 180 days following hospital admission.

The effect of AGC and TSR per hospital on care home admission and mortality was calculated for each independent variable and mutually adjusted for AGC or TSR. In addition, each hospital was labelled according to its combination of AGC rate and TSR rate resulting in 3 × 3 = 9 combinations (Fig. 1). The combination ‘AGC none—TSR low’ was excluded from the analysis due to the low number of hip fracture patients.

Multilevel binomial regression models with hospital-specific random effects were used to estimate incidence ratios with 95% confidence intervals (using the *gamm* function in the R package *mgcv*). All models were adjusted for age, sex, need for care on the day before fracture, the number of hip fracture patients/hospital/year, days from hospital admission to surgery, and medication-based comorbidity score. In all models, the continuous variables (age, number of hip fracture patients/hospital/year, medication-based comorbidity score) were standardized to have mean zero and unit variance.

Statistical analysis was performed with SAS version 9.4 (SAS Institute, Cary, NC, USA) and R version 4.0.2 (R Foundation for Statistical Computing, Vienna, Austria).

## Results

The analyses included 23,046 patients aged ≥ 80 years from 561 hospitals from 16 federal states which make up the Federal Republic of Germany. When analysing the influence of different types of geriatric treatment on outcomes on a systemic level, the three most prevalent combinations of geriatric health care after hip fracture were: rates of AGC high with TSR low; AGC medium with TSR high; and AGC medium with TSR medium (Table 2). The mean age at hospital admission was 86.7 years, 78.0% were women.

Rates of AGC were not associated with care home admission after full-adjustment for covariates. However, lower rates of AGC was associated with higher death rates (12% higher for medium rates of AGC or 20% higher for none AGC compared to high rates of AGC) (Table 3).

Treatment in hospitals with low rates of TSR was associated with a 8% higher risk of care home admission compared to treatment in hospitals with high rates of TSR (IR (95% CI) 1.08 (1.00–1.16)) after full adjustment for covariates (Table 3). Hospitals with low and medium rates of TSR were associated with higher death rates (10% and 8%, respectively) when compared to those with high rates of TSR (Table 3).

Table 4 shows associations between different combinations of rates of AGC and TSR on care home admission and death. Patients from hospitals with the combination ‘AGC medium – TSR medium’ served as reference group. The two combinations which included low rates of TSR were both associated with an increased risk of care home admission by 14% and 11%. In contrast, the combination ‘AGC high – TSR high’ was associated with an higher risk of care home admission by 20%. The combination ‘AGC none – TSR medium’ was associated with a higher death rate whilst the combination ‘AGC high – TSR low’ was associated with a reduced risk of death.

## Discussion

We analysed the association between the availability of two geriatric treatment systems used in routine medical care in Germany on care home admission and mortality in patients aged ≥ 80 years with hip fracture. AGC was not associated with care home admission but was associated with a reduction of death rate in a dose–response manner. Furthermore, low rates of TSR were associated with higher care home admission and mortality.

Notably, our results do not describe the risks or benefits for any single individual who receives AGC or is transferred to subacute rehabilitation. Since both geriatric treatment systems require a pre-defined functional status an approach which analyses the effects of AGC or subacute geriatric rehabilitation at an individual level would introduce strong selection bias. Instead, we used a systemic approach and analysed the potential benefits of the two different geriatric treatment systems according to their availability in the hospitals in which patients with hip fracture were treated. Therefore, the degree of individual benefit of receiving one or the other geriatric treatment is probably even higher than our reported estimates.

Furthermore, mortality during the acute hospital stay may affect the rate of care home admission since both are associated with comorbidity and functional disability and perhaps more importantly to be discharged to a care home the patient has to be alive [14, 15]. This is the reason why we reported mortality as an additional outcome.

Over recent years there has been growing evidence from randomized [8, 9] and large observational studies [16–20] that involvement of multidisciplinary geriatric teams in the treatment of hip fracture patients reduces mortality. In addition, meta-analyses of studies with different disease entities have shown that multidisciplinary geriatric rehabilitation is also associated with lower care home admission [21, 22]. However, the evidence is inconsistent in patients with hip fracture. One meta-analysis finds lower rates of care home admission [23], whilst another does not report a benefit [24]. Our results suggest that time and type of geriatric treatment may influence these different findings. AGC early after surgery may predominantly affect mortality, whilst subacute rehabilitation may influence care home admission and mortality. It is inconsistent to our other results that the combination ‘AGC high – TSR high’ was associated with an increased risk of care home admission. This is only insufficiently explained by a lower (not significant) death rate in this group.

A reduction of care home admission by subacute geriatric rehabilitation is also supported by another study from Germany which used historical data. It compared federal states with different geriatric treatment systems and observed the lowest rate of care home admission in the federal state with the highest rate of subacute geriatric rehabilitation [25].

Strengths of our study are the large number of included hospitals and patients, and the chosen methodical approach to analyse the scientific question on a systemic level instead of an individual level. In our opinion, this approach is robust and conservative and reduces the risk of selection bias considerably.

Care home admission is clearly influenced by personal and contextual factors. Since we used health claims data we were not able to adjust for potential confounders like cognition or social support. In addition, the analysis may have been biased by the presence of competing risks not accounted for. On the one hand, it is the nature of our observational study to analyse associations which may be influenced by the specific structure of the German health care system. Naturally, the generalisability of the study results to other countries may therefore be limited. On the other hand, AGC and subacute geriatric rehabilitation represent two basic principles of geriatric care which are present in many countries of the industrialised world.

## Conclusion

In summary, we used a ‘natural experiment’ to analyse the effect of two geriatric treatment systems on care home admission and mortality in patients aged ≥ 80 years following hip fracture. Our study demonstrated potential effects of geriatric care: lower mortality rates in hospitals with high rates of acute geriatric care and lower rates of care home admission and mortality in hospitals with high transfer rates to subacute rehabilitation.

**Table 1:** Characteristics of the two geriatric treatment systems for hip fracture patients in Germany

	**Acute geriatric care (AGC)**	**Subacute rehabilitation**
Start	Soon after surgery	Usually 7 to 14 days after surgery
Time period	At least 14 days	3 weeks, sometimes extended by 1 or 2 weeks
Orthogeriatric comanagement	Yes	no
Procedure code	OPS8-550	--
Place	Acute clinic. The orthogeriatric comanagement is delivered on an orthopedic or a geriatric unit. In Germany, common models are either shared responsibility on an orthogeriatric unit or a geriatric liaison service on the orthopedic unit with an early transfer to a geriatric unit	Subacute rehabilitation clinic
Transfer to the treatment place required	no	yes
Application at and approval by the health insurance required	no	yes
Multidisciplinary geriatric team headed by a geriatrician	yes	yes
Comprehensive geriatric assessment	yes	yes
Treatment	At least 20 units of therapy usually delivered as individual therapies within 14 days	2-4 individual or group therapies per day over 3 or more weeks.

**Table 2:** Characteristics of hospitals and patients with hip fracture aged ≥ 80 years

Hospital characteristics Number of hospitals (*N* = 561) *Number of patients (N* = *23,046)*		Rate of transfers to subacute rehabilitation (TSR)^b^		
		Low	Medium	High
**Rate of acute geriatric care (AGC)**^**a**^	**None**	5 (0.9%) *111 (0.5%)*	68 (12.1%) *2458 (10.7%)*	70 (12.5%) *2597 (11.3%)*
	**Medium**	47 (8.4%) *1798 (7.8%)*	71 (12.7%) *3552 (15.4%)*	92 (16.4%) *5008 (21.7%)*
	**High**	136 (24.2%) *4852 (21.1%)*	47 (8.4%) *1655 (7.2%)*	25 (4.5%) *1015 (4.4%)*
**Patient characteristics**				
Age (years); Mean (SD)			86.7 (4.5)	
80–85; N (%)			10,143 (44.0%)	
≥ 86; N (%)			12,903 (56.0%)	
Female; N (%)			17,973 (78.0%)	
Care need at admission; N (%)			13,106 (56.9%)	
Medication-based comorbidity score; Mean (SD)			4.0 (2.0)	
Days from hospital admission to surgery				
0; N (%)			7582 (32.9%)	
1; N (%)			9590 (41.6%)	
2; N (%)			2403 (10.4%)	
≥ 3; N (%)			1618 (7.0%)	
NA^c^; N (%)			1853 (8.0%)	

^a^Rate of acute geriatric care (OPS 8–550): None: 0%; Medium: > 0 to ≤ 48.8%; High: > 48.8%

^b^Rate of transfers to subacute rehabilitation: Low: < 18.6%; Medium: 18.6 to 43.6%; High: > 43.6%

^c^NA = No surgery-relevant OPS-code found on individual patient level data

*SD* standard deviation

**Table 3:** Associations of rates per hospital of ‘acute geriatric care’ (AGC) and of ‘transfers to subacute geriatric rehabilitation’ (TSR) with care home admission and death in patients with hip fracture aged 80 years and older

	**Care home admission**			**Death**		
**Rate of acute geriatric care (AGC)**^**a**^	**n (%)**	**IR (95% CI)**^**c**^	**IR (95% CI)**^**d**^	**n (%)**	**IR (95% CI)**^**c**^	**IR (95% CI)**^**d**^
-None	1029 (19.9)	0.94 (0.87–1.00)	0.99 (0.91–1.07)	1061 (20.5)	**1.16 (1.08–1.24)**	**1.20 (1.11–1.31)**
-Medium	2145 (20.7)	0.95 (0.90–1.01)	0.99 (0.93–1.06)	2013 (19.4)	**1.08 (1.02–1.15)**	**1.12 (1.05–1.20)**
-High	1644 (21.9)	1.00 (reference)	1.00 (reference)	1325 (17.6)	1.00 (reference)	1.00 (reference)
**Rate of transfer to subacute rehabilitation (TSR)**^**b**^			**IR (95% CI)**^**e**^			**IR (95% CI)**^**e**^
-Low	1549 (22.9)	**1.08 (1.02–1.15)**	**1.08 (1.00–1.16)**	1272 (18.8)	1.01 (0.94–1.08)	**1.10 (1.02–1.19)**
-Medium	1544 (20.1)	0.96 (0.91–1.02)	0.96 (0.90–1.02)	1571 (20.5)	**1.07 (1.01–1.14)**	**1.08 (1.02–1.15)**
-High	1725 (20.0)	1.00 (reference)	1.00 (reference)	1556 (18.1)	1.00 (reference)	1.00 (reference)

^a^Rate of acute geriatric care (OPS 8–550): None: 0%; Medium: > 0 to ≤ 48.8%; High: > 48.8%

^b^Rate of transfers to subacute rehabilitation: Low: < 18.6%; Medium: 18.6 to 43.6%; High: > 43.6%

*IR* Incidence ratio accounting for clustering of patients within hospital by multi-level modelling, *CI* Confidence interval

^c^Adjusted for age, sex, care need the day before the fracture, number of hip fracture patients/hospital/year, days from hospital admission to surgery, and medication-based co-morbidity score

^d^Adjusted for age, sex, care need the day before the fracture, number of hip fracture patients/hospital/year, days from hospital admission to surgery, medication-based co-morbidity score, and frequency category of patients per hospital transferred to subacute rehabilitation

^e^Adjusted for age, sex, care need the day before the fracture, number of hip fracture patients/hospital/year, days from hospital admission to surgery, medication-based co-morbidity score, and frequency category of patients per hospital with acute geriatric care

**Table 4:** Associations of the combination of rates per hospital of ‘acute geriatric care’ (AGC) and ‘transfers to subacute geriatric rehabilitation’ (TSR) on care home admission and death in patients with hip fracture aged 80 years and older

**Care home admission** **n (%)** **IR (95% CI)**^**c**^		**Rate of transfers to subacute rehabilitation (TSR)**^**b**^		
		**Low**	**Medium**	**High**
**Rate of acute geriatric care (AGC)**^**a**^	**None**	^d^	496 (20.2) 1.02 (0.92–1.12)	492 (18.9) 1.00 (0.90–1.10)
	**Medium**	419 (23.3) **1.14 (1.03–1.27)**	724 (20.4) 1.00 (reference)	1002 (20.0) 1.04 (0.95–1.13)
	**High**	1089 (22.4) **1.11 (1.02–1.21)**	324 (19.6) 0.99 (0.88–1.11)	231 (22.8) **1.20 (1.06–1.37)**
**Death** **n (%)** **IR (95% CI)**^c^		**Rate of transfers to subacute rehabilitation (TSR)** ^**b**^		
		**Low**	**Medium**	**High**
**Rate of acute geriatric care (AGC)**^**a**^	**None**	^d^	557 (22.7) **1.13 (1.03–1.24)**	490 (18.9) 1.00 (0.91–1.11)
	**Medium**	400 (22.2) 1.11 (1.00–1.23)	719 (20.2) 1.00 (reference)	894 (17.9) 0.93 (0.86–1.02)
	**High**	858 (17.7) **0.91 (0.83–0.99)**	295 (17.8) 0.91 (0.80–1.02)	172 (16.9) 0.93 (0.80–1.07)

^a^Rate of acute geriatric care (OPS 8–550): None: 0%; Medium: > 0 to ≤ 48.8%; High: > 48.8%

^b^Rate of transfer to subacute rehabilitation: Low: < 18.6%; Medium: 18.6–43.6%; High: > 43.6%

^c^*IR* Incidence ratio accounting for clustering of patients within hospital by multi-level modelling; Adjusted for age, sex, care need the day before the fracture, number of hip fracture patients/hospital/year, days from hospital admission to surgery, and medication-based co-morbidity score. *CI* Confidence interval

^d^The combination ‘none inpatient rehabilitation – low transfers to subacute rehabilitation’ was excluded from the analysis due to the low number of hip fracture patients

## Data Availability

Data cannot be shared publicly because they were originated from the routine data.

## Abbreviations

AOK: “Allgemeine Ortskrankenkasse”
WIdO: ”Wissenschaftliches Institut der AOK”
AGC: Acute geriatric care
TSR: Transfer to a subacute rehabilitation clinic
